# Ultra-High-Speed Image Signal Accumulation Sensor

**DOI:** 10.3390/s100404100

**Published:** 2010-04-23

**Authors:** Takeharu Goji Etoh, Dao Vu Truong Son, Toshiaki Koike Akino, Toshiro Akino, Kenji Nishi, Masatoshi Kureta, Masatoshi Arai

**Affiliations:** 1 Graduate School of Science and Engineering, Kinki University, Higashi-Osaka, Osaka 577-8502, Japan; E-Mail: best2010@civileng.kindai.ac.jp; 2 School of Engineering and Applied Sciences, Harvard University, 33 Oxford Street, Cambridge, MA 02138, USA; E-Mail: koike@seas.harvard.edu; 3 School of Biology-Oriented Science and Technology, Kinki University, Wakayama 649-6493, Japan; E-Mail: akino@info.waka.kindai.ac.jp; 4 Kinki University Technical College, Kumano, Mie 519-4935, Japan; E-Mail: nishi@ktc.ac.jp; 5 Nuclear Sensing Research Group, Nuclear Science and Engineering Directorate, JAEA, Tokai, Naka, Ibaraki 319-1195, Japan; E-Mail: kureta.masatoshi@jaea.go.jp; 6 Neutron Science Section, Materials & Life Science Division, J-PARC Center, Tokai, Naka, Ibaraki 319-1195, Japan; E-Mail: masatoshi.arai@j-parc.jp

**Keywords:** signal accumulation, averaging technique, CCD, CMOS, high speed, image sensor, ISIS, ISAS

## Abstract

Averaging of accumulated data is a standard technique applied to processing data with low signal-to-noise ratios (SNR), such as image signals captured in ultra-high-speed imaging. The authors propose an architecture layout of an ultra-high-speed image sensor capable of on-chip signal accumulation. The very high frame rate is enabled by employing an image sensor structure with a multi-folded CCD in each pixel, which serves as an *in situ* image signal storage. The signal accumulation function is achieved by direct connection of the first and the last storage elements of the *in situ* storage CCD. It has been thought that the multi-folding is achievable only by driving electrodes with complicated and impractical layouts. Simple configurations of the driving electrodes to overcome the difficulty are presented for two-phase and four-phase transfer CCD systems. The *in situ* storage image sensor with the signal accumulation function is named Image Signal Accumulation Sensor (ISAS).

## 1. Introduction

An ultra-high-speed video camera can be realized by using “In-pixel Storage Image Sensor” with pixels, each equipped with a plurality of memory elements. The very high frame rate is achieved by storing successive image signals simultaneously in the in-pixel memories in all pixels. The frame rate is the inverse of the frame interval, which is equal to the time for a charge packet to be transferred from a photodiode to a memory element in a pixel. Since the transfer time is several nanoseconds, the frame rate can theoretically reach more than one-hundred mega frames per second (>100 Mfps).

**Figure 1.**
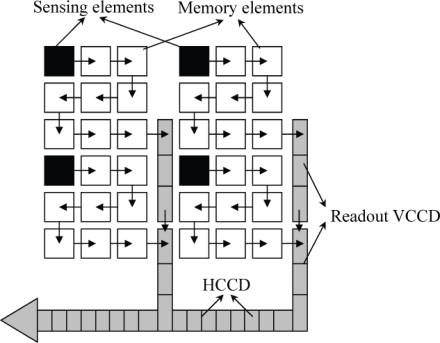
An example of a folded in-pixel CCD memory structure proposed by Elloumi *et al.* [2].

The in-pixel storage is a classic idea for ultra-high-speed imaging. For example, Morimoto in 1991 [1] and Elloumi *et al.* in 1994 [2] proposed use of a folded CCD as the in-pixel memory device, as one example shows in Figure 1. However, it has been thought that the many direction changes at folds of the folded CCD are only achievable by a complicated and impractical configuration of driving electrodes.

**Figure 2.**
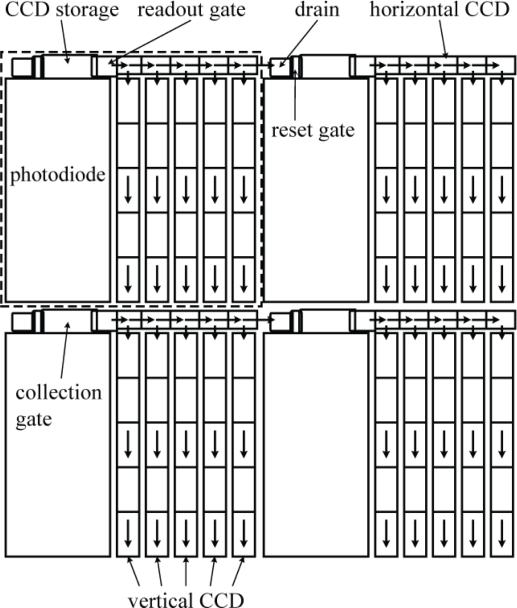
Burst image sensor by Kosonocky *et al.* [3].

In 1996, Kosonocky *et al.* developed an ultra-high-speed image sensor [3]. To overcome the difficulty in multi-transfer-direction changes, each pixel of the sensor is equipped with an SPS (Series-Parallel-Series) CCD register, which changes the transfer direction only twice; horizontally first, and then vertically, and horizontally again during image capturing, as shown in Figure 2. The sensor was capable of recording 30 consecutive images at 833,000 fps. It was named “Burst image sensor”.

**Figure 3.**
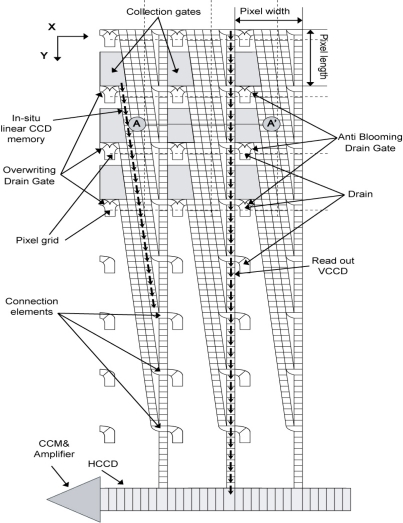
ISIS structure proposed by Etoh *et al.* [4,5].

In 2001, Etoh *et al.* developed a CCD image sensor in which a slanted linear CCD register is attached to each pixel as shown in Figure 3. It realized a simplest one-direction charge transfer with no bend. The sensor records 103 consecutive images, each with 81,120 pixels at the frame rate of 1 Mfps [4,5]. It was named “*In situ* Storage Image Sensor (ISIS)”, since each CCD storage extends through several pixels without being confined in a pixel. The ISIS camera has been extensively applied to various scientific researches, including high-speed dynamics of fluids [6], shockwaves [7], cracks [8], collision, bursting, combustion and explosion.

**Figure 4.**
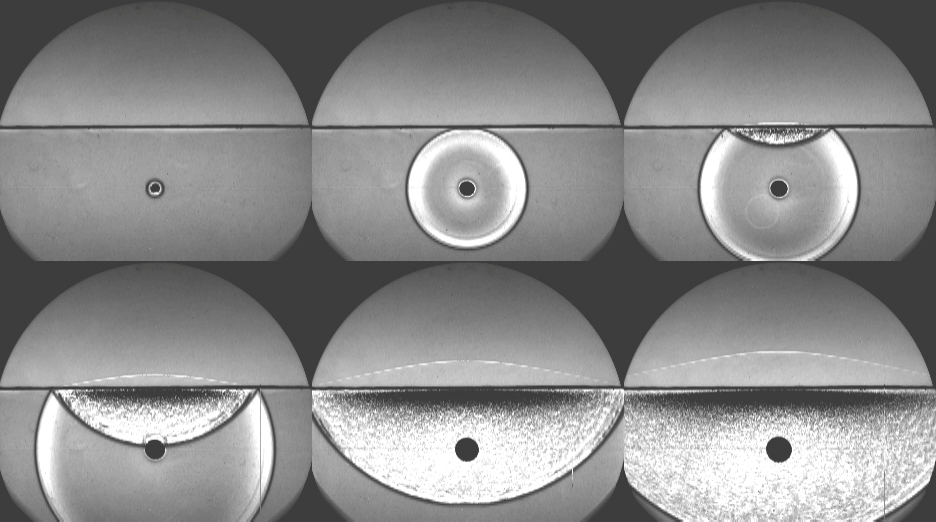
Shockwave propagation at a water surface, captured by ISIS camera [4,5] at 500,000 fps (taken by Prof. H. Kleine, University of South Wales, Australia).

Figure 4 shows an example of images of a shock wave reflected at and transferred through a water surface captured at 500,000 fps with the ISIS camera. Many important phenomena including unknown ones are observable in the images. For example, it is clearly seen that sound propagates much faster in water than in the air as learned from textbooks; the reflected wave generates a dark shadow area under the water surface and a bright mottled fan-shaped area stretching downward, which have been found later to be images of cavitation bubbles.

In ultra-high-speed imaging, there are cases in which the signal level is very low and comparable with the noise level. If the target events are reproducible, the simplest method to increase the SNR is to repeat experiments and average the accumulated data. In this paper, we propose an innovative ultra-high-speed image sensor capable of on-chip signal accumulation.

In such cases, the signal level is usually much lower than the full well capacity of the memory elements of image sensors. We have sought a structure of a CCD that makes possible *in situ* signal accumulation for a higher SNR as well as *in situ* storage for ultra-high-speed imaging. As a result, we now can finally present our image sensors with in-pixel CCDs that are folded, and yet; can be operated by driving electrodes with simple configurations.

**Figure 5.**
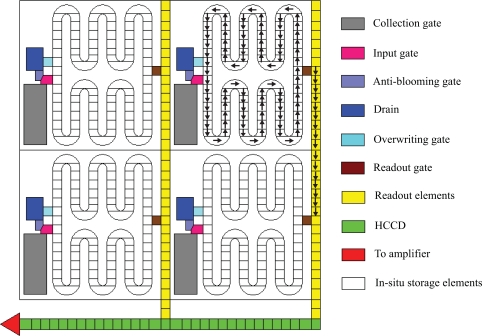
ISAS architecture on the front-side [9].

**Figure 6.**
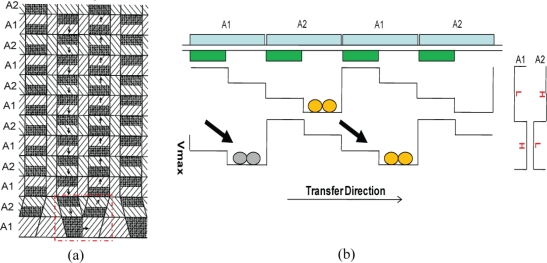
Scheme for direction change for two-phase transfer: (a) Configuration of electrodes and barriers; (b) Transfer scheme.

The general layout of the folded CCD is shown in Figure 5. The signal accumulation is realized by directly connecting the first and the last storage elements of the multi-folded *in situ* CCD. The problem is how to reverse the transfer direction with simple configurations of the electrodes. Our proposals are depicted in Figure 6 for two-phase transfer and Figure 8 for four-phase transfers in the following sections. We named the sensor “Image Signal Accumulation Sensor (ISAS)” [9].

Need of ISAS was first suggested as a detector for cutting-edge 2D-TOF (time-of-flight) neutron radiography [10]. However, we believe that the ISAS structure can benefit a much wider range of applications. In Section 4, other application examples are described, including fluorescence imaging of membrane potential of biological cells and tissues.

## 2. ISAS Structure Utilizing a Two-Phase Transfer System

### 2.1. Structure of the ISAS

We have found a very simple CCD structure that enables the transfer direction change for two-phase transfer as shown in Figure 6a. The figure shows enlargement of a folding part of the *in situ* multi-folded two-phase transfer CCD. The configuration of the electrodes and channels is as follows:

1. One CCD element consists of a channel element under a couple of alternately placed electrodes A1 and A2.
2. Under each electrode, built-in potential barriers are alternately placed on the upper and the lower halves of the neighboring CCD channels.
3. At each fold, a barrier is placed on the left half of the horizontally connecting CCD part to transfer the signal charge packet horizontally.

The operation modes for the two-phase-transfer sensor are basically the same as those for the four-phase transfer sensor, which are explained in the next section.

The configuration of the two-phase CCD system is seemingly simple. However, the transfer efficiency is delicately affected by the design, especially at the complicated parts, such as an input gate and a drain. The four-phase system much more stably transfers the signal charge packets. The two-phase and the four-phase systems both have advantages and disadvantages (see Appendix 2). The transfer scheme is selected by considering both requirements from applications and restrictions from available processes.

### 2.2. Simulation Result at a Fold of the Folded CCD Storage

**Figure 7.**
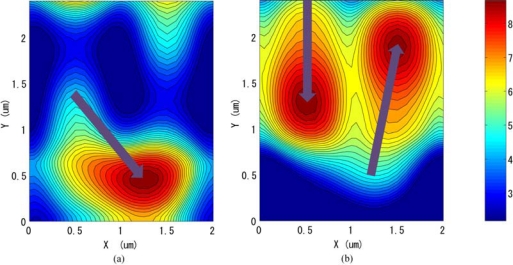
Channel potential profile at a fold of a two-phase transfer ISAS. Each memory element has a size of 1.0×3.2*μm*^2^. Arrows indicate the proposed reverse transfer direction along the fold.

Simulated channel potentials at a typical fold marked by a dotted red rectangle in Figure 6a are shown in Figure 7 at two different conditions. We can see that signal charge packet can be successfully transferred through the fold when the gates are biased at −4V to 4V alternatively.

## 3. ISAS Structure Utilizing a Four-Phase Transfer System

### 3.1. Structure

**Figure 8.**
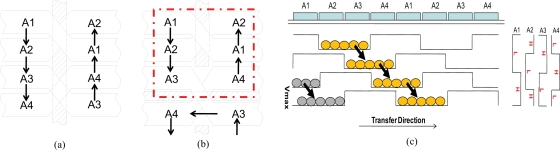
Scheme for direction change for four-phase transfer: (a) Twisted electrodes for opposite direction four-phase transfer; (b) Horizontal direction change; (c) Four-phase transfer scheme with 2-by-2 duty cycle.

We propose a configuration with “a pair of twisted double polysilicon electrodes” for a four-phase transfer CCD with folds as shown in Figures 8a and b. At each fold, a horizontal channel stop in the middle of the pair of twisted electrodes separates the upper and the lower CCDs facing each other; the vertical channel stops have open spaces to transfer charge packets in the horizontal direction. This is the key idea for folding a CCD to fit it within a pixel.

**Figure 9.**
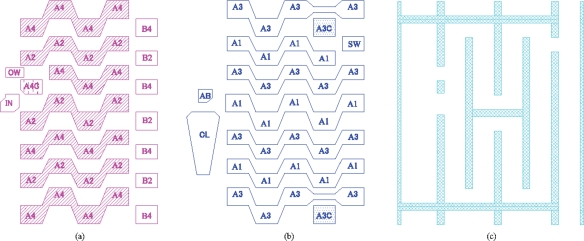
Layouts of a four-phase CCD-ISAS of: (a) the first polysilicon layer; (b) the second polysilicon layer; (c) the channel stoppers. For abbreviations, see Appendix 4.

**Figure 10.**
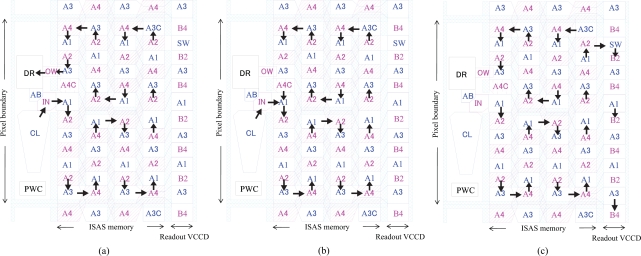
Operation schemes of CCD-ISAS: (a) Continuous overwriting mode; (b) Signal accumulation (ISAS) mode; (c) Readout mode. For abbreviation, see Appendix 4.

A whole pixel layout is shown in Figure 9 and 10 for a pure CCD sensor with a vertical readout CCD. Figure 12 shows the layout for a hybrid CCD and CMOS sensor with CCD storage and CMOS readout circuitry. Since the pure CCD-ISAS and the hybrid CCD/CMOS-ISAS have similar layouts and operation schemes, the whole structure of the CCD-ISAS is explained below with the basic operation scheme.

The folded storage CCD and the vertical readout CCD respectively consist of twelve and three CCD elements.

### 3.2. Gates and Their Basic Operations

Signal electrons generated by incident light are collected in a collection gate CL, and transferred to an input gate IN, and then to the first element of the storage CCD. If the charge packet is too large, excessive charge is drained through an anti-blooming drain gate AB to a drain DR.

The charge packet transferred on the folded storage CCD finally reaches the last CCD element neighboring the first element.

If the voltage of a gate A4C below the last CCD element is fixed at a low level, it serves as a barrier gate to prevent the charge packet from moving downward to the first CCD element. In the case, the voltage of the overwriting gate OW is fixed at a high level to continuously drain old signals to the drain DR.

On the other hand, if the voltage of OW is low and the A4C gate operates as a usual A4 gate, the charge packet is transferred downward.

A vertical readout CCD consists of a readout gate SW, the transfer gates A1 and A3, which are commonly used for the folded storage CCD and additional transfer gates B2 and B4. It is shown elsewhere [4,5] that a pair of four-phase CCDs are independently operated if two transfer gates are common and the other two are independently operated for each CCD. During readout, the transfer gate A3C serves as a barrier gate by fixing the voltage at a low level value.

### 3.3. Operation Modes of CCD-ISAS

For image capturing, the proposed ISAS is operated either in the continuous image capturing mode or the signal accumulation mode. After cease of image capturing, the readout operation mode is applied.

(1) Continuous overwriting mode

During image capturing, the OW gate is always at a high level so that old image signals are continuously drained out of the sensor via an OW-DR path. Both the A3C and A4C gates operate as normal A3 and A4 electrodes, respectively. The latest image signals are always successively stored in the *in situ* storage. The operation continues in all pixels until a target event occurs and a trigger signal is released to stop the overwriting recording operation.

(2) Signal accumulation mode

For cyclic signal accumulation mode, signals stored in the *in situ* memory obtained in a previous experiment are added to new signals obtained in a current experiment, when they are transferred through the A4C gate. Both the A3C and A4C gates operate as normal A3 and A4 electrodes, respectively. The operation repeats many times to accumulate signals. If the accumulated signal size becomes larger than the full well capacity, the exceeding amount is drained through the OW gate.

(3) Readout mode

To read out the image signals stored in the storage CCD, three signals are transferred from the storage CCD to the readout CCD to fill the readout CCD with signals. The nine remaining signals are still kept in the storage CCD and the three CCD elements of the storage CCD are now empty. After transferring the signals in the vertical readout CCDs to a horizontal readout CCD (not drawn) and, then, to the readout amplifier, the vertical readout CCD becomes empty. By repeating the process four times, all image signals stored in the storage CCDs in all pixels are read out to a buffer memory device outside the image sensor.

Depending on image signal levels, the sensor can be operated with or without signal accumulation. If the signal level is sufficiently high in comparison to the noise level, signals stored in the *in situ* storage can be immediately read out after cease of image capturing operation. Otherwise, signal accumulation operation is necessary to increase the signal-to-noise ratio.

For ISAS fabricated by a pure CCD process, only a sequential readout operation is available. However, in section 3.7, we will show that it is possible to employ other readout modes by integrating a CMOS readout circuitry in each pixel.

### 3.4. Simulation Result at a Fold of the Folded CCD Storage

**Figure 11.**
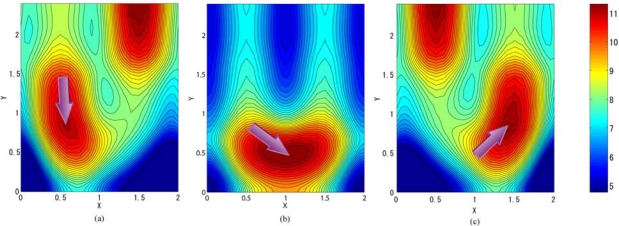
Channel potential profile at a fold of a four-phase transfer ISAS. Each memory element has a size of 1.0×3.2*μm*^2^. Arrows indicate the proposed reverse transfer direction along the fold.

Simulated channel potentials at a typical fold marked by a dotted red rectangle in Figure 8b are shown in Figure 11 at three different conditions. Utilizing a standard 50% duty cycle four-phase transfer, it is easy to reverse transfer direction at the fold. For example, in Figure 11a, A2 and A3 gates are biased at a high level (+4V), the charge signal is stored under A2 and A3 gates; when A2 and A4 are respectively switched to a low level (−4V) and a high level (+4V) as in Figure 11b, the signal charge is transferred to the adjacent electrodes.

### 3.5. Frame Rate Limitation of the ISAS

As we move toward a higher working frame rate, parasitic properties of wires, interconnections, and buried channel memories cause a drop of the driving voltage, which seriously decreases the maximum frame rate [11]. Similar challenges are also being reported in the development of fast-framing imaging systems utilizing CCD sensors for the vertex detector and mass spectrometry [12–14].

We proposed the following practical countermeasure to the problem:

1. Employ a BSI (Backside Illumination) structure, and
2. Place crossed differential buslines on the top of the front surface by using a couple of additional metal layers.

The countermeasure effectively reduces both the resistance and the reactance, and serves to achieve the maximum frame rate up to 100 Mfps [11]. Currently, advanced CMOS imager process utilizes four or more metal layers. We can allocate the top two metal layers for the crossed differential buslines.

### 3.6. Expected Noise Performance

The proposed ISAS has *in situ* CCD memories. Theoretically, a CCD can be operated without noise, if sufficiently cooled down. Therefore, the major noise source of the ISAS is the readout noise. Due to the local *in situ* storage structure, the ISAS can be operated at a very slow rate during readout operation, which effectively reduces the readout noise, too. Incorporation of these advantageous characteristics of the ISAS with averaging of accumulated signals and backside illumination, the noise level associated with the averaged signals can be reduced down to less than a single-photon level.

As explained above, the ISAS provides extremely high SNR for imaging of reproducible events.

It is worth noting that the noise level of the ISAS is independent of the frame rate during image capturing operations without a readout operation, since the major noise source is the readout noise.

### 3.7. Hybrid CCD/CMOS-ISAS

**Figure 12.**
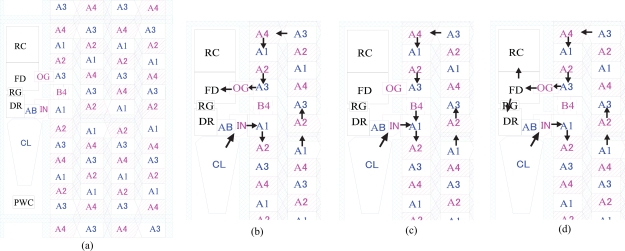
Operation of Hybrid CCD/CMOS ISAS: (a) Plan of a pixel; (b) Continuous overwriting mode; (c) Signal accumulation (ISAS) mode; (d) Readout mode. For abbreviation definitions, see Appendix 4.

The hybrid CCD/CMOS-ISAS differs from the CCD-ISAS only in that a floating diffusion amplifier and a CMOS readout circuit are installed in the top left corner of each pixel area just above the drain area as shown in Figure 12. The circuit design is similar to that of a standard CMOS active pixel sensor. However, it is worth noting that the FD is also being used as a drain during continuous overwriting mode.

Incorporating CMOS readout technology with a storage CCD enables us to obtain an ultimate high speed image sensor. The CMOS technology provides the flexible and high speed readout; the CCD technology provides the ultra-high-speed, signal accumulation and superior image quality.

## 4. Potential Applications

One promising application target is high speed membrane potential imaging of living cells and tissues of the central nervous system with voltage sensitive dyes (VSD) [14]. Available VSDs on the market provide fractional changes of fluorescent intensity of up to a few percent during the occurrence of firing action potentials lasting only a few milliseconds. Scientists and engineers always have to perform averaging technique for a plurality of high speed imaging experiments to obtain more recognizable data.

Our original application target was a cutting-edge 2D-TOF (time-of-flight) neutron radiography [10] (Appendix 1). Development of the ISAS was first suggested by the fifth and sixth author. They require an image sensor with both very high spatial and temporal resolutions. Furthermore, the incident light is very weak, also requiring very high sensitivity. Fortunately, since events in the experiments are reproducible, a signal accumulation and averaging technique is applicable to increase the SN ratio by repeating the experiments many times.

Other possible applications include ultrasonic wave imaging of solid surfaces, high speed non-destructive tests and measurements by TEM, *etc.*

Generally, the proposed ISAS can be of great use to applications that satisfy the following conditions:

1. The target event is reproducible.
2. Light intensity is low which generates a signal electron packet much less than the full well capacity of a single storage element
3. Number of frames required is up to 100 to 200 frames.

## 5. Conclusions

The proposed image sensor has a new function to accumulate consecutive signals captured in repeated ultra-high-speed imaging of reproducible events. The sensor is named ISAS, the image signal accumulation sensor. CCD-based architecture layouts to realize ISAS are presented. The key elements of the layouts are as follows:

1. A fold of a CCD with a simple and practical configuration.
2. Connection of the first and the last elements of a topologically linear CCD.

The fold structure enables multi-folding of the *in situ* storage CCD for ultra-high-speed imaging. The connection of the head and tail elements enables signal accumulation.

For both two-phase and four-phase transfer CCD systems, the fold structures are proposed. By using the structures, example pixel layouts are presented along with explanations of the operation schemes.

An example layout of a hybrid CCD/CMOS ISIS is also proposed which enables an ultimate ultra-high-speed image sensor with ultra-high-speed and high image quality by the *in situ* CCD storage and flexible high-speed parallel readout by the CMOS readout circuitry.

Finally, some potential applications are presented.

## Appendix 1

The 2D-TOF neutron radiography is one of main functions provided by the J-PARC (Japanese Proton Accelerator Research Complex). Protons accelerated to 99.9% of light speed are injected to Mercury, which serves as the target and the refrigerant. Neutrons scattered by the collision are introduced to a deuterium pool, which release thermal neutrons. Velocity of the thermal neutrons is dependent of the energy; those with higher and lower energy levels respectively pass through the specimen earlier and later and hit a scintillator, which releases gamma rays; then, the gamma rays hit a fluorescence layer, which releases visible light. The thermal neutrons interact with different species of atoms dependent of their energy levels. Consequently, their arrival times which are detected by a high-speed video camera provide information on ingredients of the specimen. The problem is that intensity of the generated light is very weak. However, the event is reproducible. Therefore, the ISAS best fits to detect meaningful signals by repetition of the experiments.

The URL of J-PARC is as follows: http://j-parc.jp/index-e.html

## Appendix 2

The four-phase transfer system has advantages and disadvantages to the two-phase transfer system.

1. One CCD element of the four-phase system consists of a channel element with four driving electrodes. Therefore, if the size of the CCD element is the same, the width of the electrode is a half of that for the two-phase system, which requires a much finer fabrication process and causes lower DC-yield.
2. The transfer speed of the four-phase transfer is a half or a quarter of that of the two-phase transfer.
3. The charge handling capacity of the four-phase transfer is three to six times larger than that of the two-phase transfer.
4. The four-phase transfer is much more stable than the two-phase transfer. Design of image sensors with the four-phase transfer is much easier.

## Appendix 3

While folding CCD channels had been thought to be difficult, it is possible to construct ISAS for any types of CCD structures with simple configurations. For example, the three-phase CCD can be folded by twisting two of three electrodes; the single-phase CCD (virtual-phase CCD) by fixing voltage of or removing one of two electrodes of the two-phase CCD structure shown in Figure 6.

## Appendix 4

Symbols used in Figures 8, 9, 10 and 12 are as follows:

1. CL (collection gate): to collect photo-electrons generated from the backside of each pixel to the *in situ* storage on the front side.
2. IN (input gate): to transfer signal charge packets to first element of the *in situ* storage.
3. AB (anti-blooming gate): to drain excessive charges out of the sensor.
4. DR (drain): n-substrate contact.
5. A1/A2: two-phase transfer electrodes for the *in situ* storage.
6. A1/B2: two-phase transfer electrodes for the readout VCCD.
7. A1/A2/A3/A4: four-phase transfer electrodes for the *in situ* storage.
8. A1/B2/A3/B4: four-phase transfer electrodes for the readout VCCD.
9. OW (over-writing gate): to help to drain charge packets out of the sensor.
10. SW (switching gate): to transfer image signals stored in the *in situ* storage to readout VCCD.
11. PWC (p-well contact): to drain holes generated in the front side out of the sensor.
12. RG (reset gate): to control reset operation.
13. FD: floating diffusion node.
14. RC (readout circuitry): conventional CMOS readout circuitry consisting of one source follower transistor, and row and/or column select transistor(s).
